# A Low-Intensity Nutrition Intervention Targeting Triglycerides in Gestational Diabetes: A Feasibility RCT

**DOI:** 10.1210/clinem/dgaf291

**Published:** 2025-05-16

**Authors:** Kai Liu, Georgia S Clarke, Jessica A Grieger

**Affiliations:** Adelaide Medical School, The University of Adelaide, Adelaide, SA 5000, Australia; Robinson Research Institute, The University of Adelaide, Adelaide, SA 5000, Australia; Lifelong Health Theme, South Australian Health and Medical Research Institute, Adelaide, SA 5000, Australia; Adelaide Medical School, The University of Adelaide, Adelaide, SA 5000, Australia; Robinson Research Institute, The University of Adelaide, Adelaide, SA 5000, Australia; Lifelong Health Theme, South Australian Health and Medical Research Institute, Adelaide, SA 5000, Australia; Adelaide Medical School, The University of Adelaide, Adelaide, SA 5000, Australia; Robinson Research Institute, The University of Adelaide, Adelaide, SA 5000, Australia; Lifelong Health Theme, South Australian Health and Medical Research Institute, Adelaide, SA 5000, Australia

**Keywords:** pregnancy, gestational diabetes, feasibility studies, prenatal care, dietary intervention

## Abstract

**Context:**

The conventional approach to diet therapy for gestational diabetes mellitus (GDM) is carbohydrate modification to mitigate glucose-mediated fetal macrosomia. Maternal triglyceride concentrations more strongly relate to infant adiposity than maternal glucose.

**Objective:**

This work aimed to assess the feasibility of a low-intensity dietary intervention designed to attenuate the rise in triglycerides compared to standard GDM management.

**Methods:**

Women with GDM were randomly assigned at approximately 30 weeks’ gestation to a standard care group (ie, usual GDM management) or to an intervention group, at an allocation ratio of 1:1. The intervention group received standard care plus individual counseling on reducing intake of ultraprocessed foods, increasing fruits, vegetables, fish and nuts, and changes to healthier fats. The primary outcome is study feasibility; secondary and exploratory outcomes include maternal dietary intakes, plasma triglyceride and glucose levels, and birth weight.

**Results:**

Over 10 months of active recruitment, 444 women were invited to participate. Of these, 59 were eligible (13.2%), 38 (8.6%) consented and were randomly assigned (n = 19 intervention, n = 19 standard care), and 34 women completed the study. The recruitment rate was 1 per week, the retention rate was 89.5%, and the feasibility of eligibility criteria was 70.4%. Nearly all women in the intervention group who responded to the questionnaire (n = 15/16) reduced their ultraprocessed food intake, and 11 women increased their intake of nuts. There was no end of study differences in nonfasting plasma triglycerides (mean [95% CI] in intervention, 2.84 [2.22-3.46] mmol/L vs standard care, 3.40 [2.78-4.02] mmol/L). Mean birthweight was higher in the standard care group vs intervention group (mean difference [95% CI], 479.5 [110.7-848.3] g).

**Conclusion:**

There was a modest recruitment rate and a high retention rate, indicating a diet aimed at attenuating triglycerides is feasible and highly acceptable in women with GDM. The positive improvements observed in maternal diet and desirable birth weight warrant further investigation in a larger, definitive, randomized controlled trial.

Gestational diabetes mellitus (GDM) is hyperglycemia first detected in pregnancy, with a pooled global standardized prevalence of 14.0% (1). Women with GDM have an 8-fold increased risk for developing later type 2 diabetes (2) and a 2-fold increased risk of cardiovascular diseases (3). Hyperglycemia in pregnancy is associated with increased fetal growth (4, 5), along with higher rates of obesity, impaired glucose tolerance, and hypertension in childhood and adolescence (6).

The conventional approach to diet therapy for GDM is carbohydrate modification toward high-fiber sources, with the goal of blunting postprandial glucose, to mitigate glucose-mediated fetal macrosomia (7). Glucose is the most important nutrient crossing the placenta and is key to fetal development (8). However, excess glucose following high-carbohydrate intake contributes to maternal hyperglycemia and insulin resistance (9). Problematically, lower-carbohydrate diets typically result in higher fat intake, given protein intake does not significantly change (10). A high-fat diet, particularly one that is high in saturated fat, increases inflammation and oxidative stress, and impairs skeletal muscle glucose uptake (11). In pregnancy, such a high-fat diet (in combination with the recommended and conventional lower-carbohydrate diet) may contribute to placental dysfunction (12), increased infant adiposity (13, 14), and initiation of adverse developmental programming (15).

There is currently no optimal dietary approach that effectively and consistently manages GDM. A meta-analysis of 18 randomized controlled trials (RCTs) involving 1151 women with GDM demonstrated that various dietary interventions led to a larger decrease in fasting (−0.2 mmol/L) and postprandial glucose (−0.4 mmol/L) as well as a lower need for medications (relative risk, 95% CI: RR 0.65; 95% CI, 0.47-0.88) compared to standard care. For infant outcomes (16 RCTs, n = 841), modified dietary interventions were associated with lower infant birth weight (−170 g; −333, −8) and less macrosomia (RR 0.49; 0.27, 0.88), but not large for gestational age (16). However overall, the sample sizes of the included studies were small (range, 12-150), the quality of evidence was low to very low, and the dietary interventions were heterogeneous.

In normal pregnancy, triglycerides, which are derived from the diet or endogenously produced by the liver from fatty acids, increase (17). We (18, 19), and others (20, 21), have shown triglycerides are a strong risk factor for GDM, and increased triglycerides are the most important metabolic factor in GDM, explaining more of the variance than conventional risk factors such as blood glucose, maternal age, and family history of diabetes (22). Additionally, maternal triglycerides are predictors of macrosomia, independent of maternal body mass index (BMI) and glycemic control (23, 24). Although triglyceride concentrations can be influenced by diet, there are minimal dietary intervention studies identifying key foods/food groups that reduce triglycerides (25), and none have been conducted in women with GDM, which may be important for improved fetal growth outcomes. Based on our simulation modeling, which estimated the effects of manipulating dietary changes on triglycerides in reproductive-aged women (26), we now test the optimal diet in a pilot study. The aim of this pilot study is to assess the feasibility of a low-intensity dietary intervention designed to attenuate the rise in triglycerides compared to standard GDM management.

## Materials and Methods

### Study Design

This was a parallel-group, feasibility, RCT in pregnant women diagnosed with GDM. The study was registered at the Australian and New Zealand Clinical Trial Registry (ACTRN12623000199617p), and ethics approval was obtained by the Women's and Children's Health Network Human Research Ethics Committee (2023/HRE00008). The Consolidated Standards of Reporting Trials (CONSORT) reporting guidelines were followed.

### Participant Recruitment and Eligibility

Women were recruited by one of two strategies: 1) from the GDM group education session provided at the Women's and Children's Hospital between May 10, 2023 to August 2, 2024; or 2) through paid Facebook and Instagram advertisements on the University of Adelaide social media platforms between July 18, 2023 to May 31, 2024.

Women who registered interest were sent an online screening questionnaire to assess their eligibility that included English speaking, aged older than 18 years, and current GDM diagnosis. In Australia, GDM is diagnosed through an oral glucose tolerance test usually performed between 24 and 28 weeks’ gestation, using the International Association of Diabetes in Pregnancy Study Groups/Australasian Diabetes in Pregnancy Society diagnostic criteria (27). That is, plasma blood glucose levels are greater than 10.0 mmol/L at 1 hour and/or 8.5 to 11.0 mmol/L 2 hours post 75-g oral glucose challenge. In Australia, women may be scheduled for an earlier oral glucose tolerance test before 20 weeks of gestation if they are identified to be higher risk, for example, maternal age of 40 years or older, polycystic ovary syndrome, previous hyperglycemia in pregnancy, or family history of diabetes (28). Exclusion criteria was overt diabetes in pregnancy, type 1 or type 2 diabetes, poorly controlled hypothyroidism, Graves disease, multiple pregnancy, vegetarian or vegan diet, and use of steroids, lipid-lowering drugs, or antipsychotics, or antibiotic use in the past 3 months.

### Randomization

Eligible women who provided informed consent were randomly assigned at a 1:1 ratio to standard GDM care or the intervention group (ie, standard GDM care + dietary intervention) using permuted block randomization within a web-based service, REDCap. Randomization was performed without stratification due to the small sample size and the primary aim of assessing feasibility. Neither the study coordinators nor participants were blinded to the group allocation after randomization.

### Study Protocol

**Figure 1.**
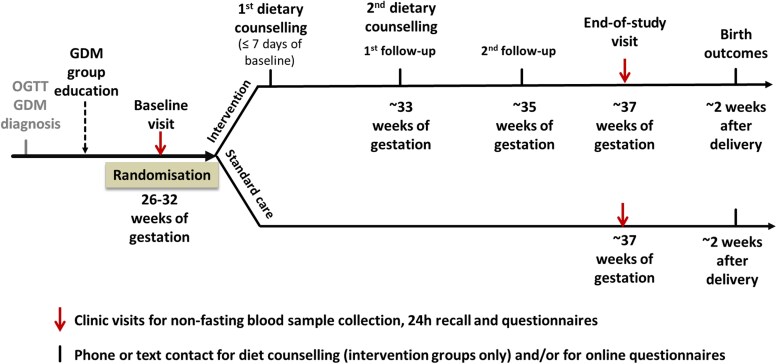
Study timeline.

Eligible women attended the research clinic at baseline (26-32 weeks’ gestation) and end of study (as close as possible to their estimated delivery date) at the Women's and Children's Hospital or at the South Australian Health and Medical Research Institute. The study timeline is presented in Fig. 1. At each visit, women completed a food questionnaire and a 5-step, 24-hour dietary recall (29) to collect dietary information. Nonfasting venous samples were collected in EDTA and fluoride oxalate vacuum tubes, centrifuged immediately (1500 rpm, 10 minutes, 4 °C) and frozen at −80 °C on site. For each participant, blood samples were drawn at similar times of day at each visit. Time of last food intake before blood collection was recorded at the clinic. The baseline visit also included a series of questionnaires to collect demographic data (including ethnicity, education level, and household income), and pregnancy-related data (including maternal age, BMI, gravidity, parity, alcohol drinking and smoking during pregnancy, as well as supplementation and medication use). Self-reported prepregnancy weight was collected, and height was measured using a stadiometer at the research clinic. At the end of the study, women from the intervention group also completed a study feedback questionnaire. Two weeks following delivery, women were contacted to complete questionnaires for birth outcomes and to upload a photo of their infant birth page in the standard My Health and Development Record used in Australia.

### Dietary Intervention

#### Standard care group

All women received usual GDM care, which includes conventional diet therapy focused on carbohydrate modification, self-blood glucose monitoring, and physical activity, with follow-up appointments when required (28).

#### Intervention group

In addition to standard GDM care, women randomly assigned to the intervention group received 2 individual dietary counseling sessions (a 30-minute face-to-face session or phone call) with the study dietitian. The initial session was scheduled within 7 days of the baseline clinic visit (26-32 weeks). Dietary advice was provided on food choices to select or modify, based on our previous modeling work to alter triglycerides through dietary choices (26). The dietary session included practical advice on how to reduce various ultraprocessed foods by half (as gram weight), or to completely replace certain ultraprocessed foods with minimally processed dietary options. Processed/ultraprocessed food items were identified according to the NOVA food classification system (30). Participants were also advised to increase intakes of whole fruits, vegetables, fish high in ω-3 fatty acids (eg, salmon, trout, sardines), nuts, and potential changes to high saturated fat spreads or oils. Dietary strategies were personalized according to their habitual intake identified from their baseline food questionnaire and 24-hour recall. Care was taken to ensure advice was culturally relevant and appropriate. At approximately 30 to 33 weeks’ gestation (the first follow-up and approximate mid-point of the study), women were contacted through text messages for self-reported adherence and satisfaction to the intervention, using a 10-point Likert scale (a score of 10 means higher adherence or satisfaction). Based on the adherence check, women had a phone call review session with the study dietitian, who provided further dietary support including behavior change techniques such as goal setting, counterconditioning, reinforcement management, and problem solving. No further dietary support was provided after this phone review. Dietary adherence was assessed again 2 weeks later both at the second follow-up (32-35 weeks’ gestation) through text messages, and at the end of study clinic visit (∼37 weeks’ gestation).

### Primary Outcome

#### Study feasibility

Feasibility was assessed by 1) recruitment rate, defined as the average number of participants recruited and randomly assigned per month; 2) participant retention rates, calculated as the percentage of randomly assigned participants who were assessed and included in the primary outcome analysis (ie, the number of participants included in the analysis of the primary outcome divided by the number of participants recruited and randomly assigned); and 3) feasibility of the eligibility criteria (consent rate), calculated as the percentage of eligible participants who consented and were randomly assigned (ie, the total number of participants recruited and randomly assigned divided by the number of eligible participants). As this is a pilot, exploratory study, no a priori power calculation was undertaken; however, previous pilot trials (31-35) consider n greater than 12 per group adequate to assess feasibility, adherence, and compliance. The recruitment was conducted in the planned period between May 10, 2023 to Aug 2, 2024, aiming for a minimum of 12 women randomly assigned to each group.

### Secondary Outcomes

To address potential challenges in study design and methodology, two secondary outcomes were included: 1) the feasibility of collecting an additional nonfasting blood sample at baseline and end of study. This was calculated as the proportion of women who did not have a blood sample taken divided by the total number of participants and reported as percentage. Reasons for missing nonfasting blood samples at baseline and end of study were recorded. 2) Participant adherence to the dietary intervention using a 10-point Likert scale (a score of 10 means higher adherence or satisfaction). This self-report was assessed in the intervention group only at the first and second follow-up and at the end of study clinic visit.

### Exploratory Outcomes

#### Dietary intake

Frequencies of intake of key food groups (fruit, vegetables, nuts, oils/spreads, and ultraprocessed foods) were collected using food questionnaires administered at baseline and at the end of the study. Total daily energy intake, including protein, carbohydrates (including sugar, added sugar, and dietary fiber), and fat (including saturated, monounsaturated and polyunsaturated fatty acids, linoleic acid, α-linoleic acid, and very-long-chain ω-3 fatty acids), was assessed based on the single 24-hour recall at baseline and at the end of the study. Diet data were analyzed using the FoodWorks10 professional program (version 10.0.4266, Xyris Software).

#### Plasma biomarkers

Baseline and end of study plasma triglycerides and glucose were measured using commercially available enzymatic kits on an automated clinical analyzer (Indiko Plus, Thermo Fisher Scientific).

#### Birth outcomes

Gestational weeks at delivery, mode of delivery, birth weight, and 5-minute APGAR score were collected from the birth outcome questionnaire and cross-checked with the uploaded birth page photo. Infants born with a greater than 4000-g birth weight were reported as macrosomia. Small and large for gestational age (≤10th and ≥90th percentile, respectively) were assessed using the GROW centile calculator (GROW Centile Calculator, version 2.1.6.1).

### Statistical Analysis

Study feasibility (recruitment and retention rate), and secondary outcomes were reported descriptively. Exploratory measures for plasma triglyceride and glucose concentrations, dietary intake (energy, nutrients, and food groups), and birth weight are reported by treatment group as mean (SD or 95% CI) or median (interquartile range, IQR) for continuous outcomes, and n (%) for categorical outcomes, unless otherwise specified. An unadjusted linear regression model was used to test the effect of the intervention compared to standard care on end of study plasma triglycerides, glucose, macronutrient intakes, gestational weeks at delivery, and birth weight. Data were analyzed using SPSS (version 29, IBM Corp).

## Results

The mean ± SD age and prepregnancy BMI for the 38 women was 32.3 ± 4.7 years and 29.6 ± 8.3 kg/m^2^, respectively. Just over half of the women self-reported to be White (52.6%), followed by Asian ethnicity (34.2%). Five women self-reported to be of Aboriginal or Torres Strait Islander, American, or African ethnicity. Most women (97.4%) completed at least secondary education and 78.9% were employed at the time of the study. Nearly 30% of the women were primigravida and 44.7% were nulliparous. No woman self-reported to drink alcohol or smoke during their pregnancy, whereas 23 women reported to drink alcohol during the 3 months leading up to pregnancy. Most women (71.1%) were taking supplements during pregnancy, 2 of whom were taking ω-3 fish oil supplements (both in the intervention group). Around a third of women reported to take metformin (12.2%) and/or insulin (18.4%) for management of GDM. Baseline characteristics for the intervention and standard care groups are presented in Table 1.

**Table 1.** Baseline characteristics of the study participants

	Intervention (n = 19)	Standard care (n = 19)
Age, (mean ± SD), y	33.5 ± 4.2	31.1 ± 5.0
Gestational wk at baseline (mean ± SD)	30.5 ± 1.6	30.9 ± 1.5
Prepregnancy BMI, (mean ± SD), kg/m^2^	26.9 ± 8.1	32.2 ± 7.7
Ethnicity, n (%)		
White (Australian, European)	8 (42.1%)	12 (63.2%)
Indigenous (Aboriginal or Torres Strait Islander, Maori, Native American)	0 (0%)	2 (10.5%)
Asian (North Asian, Southeast Asian, Southern and Central Asian)	10 (52.6%)	3 (15.8%)
American (North, Central, and South American)	1 (5.2%)	1 (5.2%)
African	0 (0%)	1 (5.2%)
Education level, n (%)		
Completed secondary education, yes	19 (100%)	18 (94.7%)
Completed tertiary education (undergraduate or postgraduate degrees), yes	17 (89.5%)	13 (68.4%)
Employed during pregnancy, n (%)	17 (89.4%)	13 (68.4%)
Gravidity, n (%)		
Primigravida	5 (26.3%)	6 (31.6%)
Multigravida	14 (73.6%)	13 (68.4%)
Parity, n (%)		
Nulliparous	9 (47.3%)	8 (42.1%)
Primiparous	7 (36.8%)	9 (47.3%)
Multiparous	3 (15.8%)	2 (10.5%)
Annual household income ($AUD), n (%)		
<40 000	2 (10.4%)	4 (21%)
40 001-70 000	3 (15.8%)	3 (15.8%)
70 001-105 000	5 (26.3%)	6 (31.6%)
105 001-205 000	7 (36.8%)	6 (31.6%)
>205 000	0 (0%)	0 (0%)
Prefer not to disclose	2 (10.5%)	0 (0%)
Alcohol consumption, n (%)		
3 mo leading up to pregnancy, yes	11 (57.9%)	12 (63.2%)
During pregnancy, yes	0 (0%)	0 (0%)
Smoking, n (%)		
Before pregnancy, yes	0 (0%)	1 (5.2%)
During pregnancy, yes	0 (0%)	0 (0%)
Supplementation, yes n (%)	15 (78.9%)	12 (63.2%)
Multiple micronutrients, yes n (%)	15 (100%)	12 (100%)
Medication, yes n (%)	9 (47.4%)	8 (42.1%)
Metformin	2 (22.2%)	3 (37.5%)
Insulin	4 (44.4%)	3 (37.5%)

Abbreviation: BMI, body mass index.

### Primary Outcomes

#### Recruitment efficiency and participant retention

**Figure 2.**
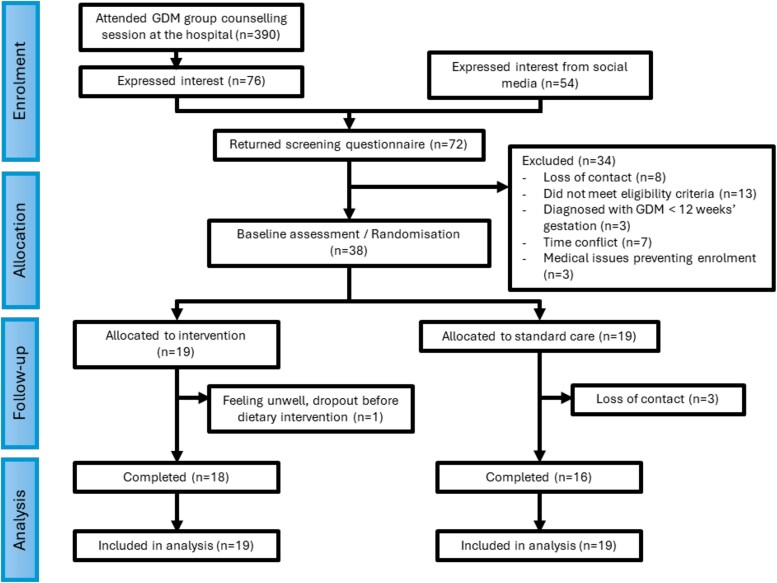
Flow diagram of participants in the study.

Over a period of 10 months (37 weeks) of active participant recruitment, 390 women attended the standard GDM group counseling sessions and received study information, of whom 76 registered their interest to participate. A further 54 women registered their interest through social media. The screening questionnaire was sent to all 130 women who were interested, and 72 women responded to the questionnaire for eligibility assessment. In total, of the 59 women deemed eligible according to the inclusion/exclusion criteria, 38 women provided informed consent and were randomly assigned to the intervention group (n = 19) or the standard care group (n = 19). The recruitment rate was approximately 4 consenting women per month (1 per week), and the feasibility of eligibility criteria was 70.4%. One woman dropped out from the intervention group before the first dietary counseling session, and 3 women from the standard care group lost contact before the end of study visit (Fig. 2). Thirty-four women completed the trial with a retention rate of 89.5%. All women attended their baseline session between 26 and 32 weeks’ gestation, and the end of study visits between 35 and 37 weeks’ gestation, with individual trial participation between 5 and 10 weeks.

### Secondary Outcomes

#### Self-reported adherence and feedback from the intervention group

The dietary intervention feedback from the 18 women in the intervention group is summarized in Table 2. Sixteen women rated the dietary advice provided by the study dietitian as “mostly relevant” (n = 11) or “very relevant” (n = 5). Most women reported to prefer short and direct dietary information (n = 16); had a preference to receive their health and dietary advice regularly (n = 15); and had a preference to seek advice only on their request (n = 14).

**Table 2.** Intervention participants’ feedback on their preferences to receiving the study-related dietary advice (n = 18 completed)

Items/questions	Yes, n (%)
1. How do you rate the relevance of the dietary advice provided to you in the study?	
Not relevant	0 (0%)
Somewhat relevant	2 (11.1%)
Mostly relevant	11 (61.1%)
Very relevant	5 (27.8%)
How would you prefer to receive your dietary feedback?	
2. Preference for information on how to obtain positive results	18 (100%)
3. Preference on how to prevent negative consequences	17 (94.4%)
4. Preference for seeking advice when the respondent requests	14 (77.8%)
5. Preference to receive advice at a routine/specific time	15 (83.3%)
6. Preference for short and direct information	16 (88.9%)
7. Preference for detailed information that includes explanations about why the advice is good for the respondent	15 (83.3%)

**Figure 3.**
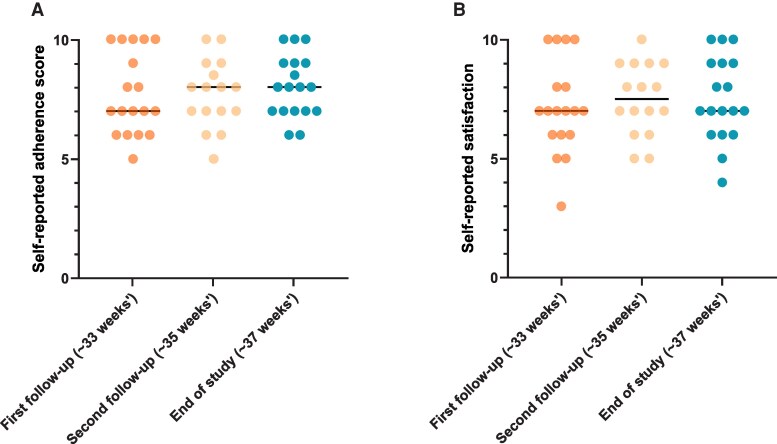
Self-reported (A) adherence to and (B) satisfaction with the dietary intervention.

The median self-reported adherence to the dietary intervention (with 10 being very adherent) increased from a score of 7 at the first follow-up to a score of 8 at the second follow-up, and this adherence score was maintained until the end of the study (Fig. 3A). Self-reported satisfaction with the dietary advice was maintained at a median score of 7 (with 10 being very satisfied) throughout the intervention period (Fig. 3B). There was an increase in dietary adherence among 8 women, whereas 5 women reported to have no change in score and 5 women became less adherent at the end of the study.

#### Feasibility of nonfasting blood sample collection

Four of the 38 randomly assigned women (10.5%) were unable to provide a blood sample at baseline, and 8 women (21.1%) had a missing blood sample at the end of the study due to loss of contact (n = 4) or going into labor (n = 4) before the scheduled clinic visits. The baseline blood samples were taken at a median of 1.7 (IQR: 0.95-2.45) hours from time of last food intake, and the end of study blood samples were collected at a median of 1.2 (IQR: 0.7-2.4) hours from time of last food intake.

### Exploratory Outcomes

#### Food and nutrient intakes

In the intervention group, a higher number of women were consuming nuts more frequently by the end of the study, that is, from less than 2 times per week to at least 3 times per week (Table 3). For ultraprocessed foods, a higher number of women reduced their intake, that is, more women were consuming ultraprocessed foods less than 1 time per week compared to this amount at baseline, and fewer women were consuming ultraprocessed foods at least 4 times per week compared to baseline. For fruit, a higher number of women were consuming fruit at least 2 times per day compared to this amount at baseline, and fewer women were consuming fruit less than 2 times per day. Intake of vegetables remained fairly consistent both for the intervention and standard care groups.

**Table 3.** Participant changes in food groups over the study period

	Baseline		1st follow-up	2nd follow-up	End of study	
	Intervention	Standard care	Intervention	Intervention	Intervention	Standard care
Fruit, n						
Never	0	0	0	1	1	0
<2 times/d	8	9	7	4	4	6
≥2 times/d	11	10	11	12	13	10
Vegetables, n*^a^*						
Never	0	0	0	0	0	0
<1 time/d	2	1	1	1	1	0
1-2 times/d	9	7	11	8	9	8
3-4 times/d	7	7	5	6	5	5
≥5 times/d	1	2	1	2	3	2
Nuts, n						
Never	0	2	0	0	0	2
<1 time/wk	3	3	1	0	0	2
1-2 times/wk	7	7	2	2	2	5
3-4 times/wk	3	4	8	7	6	5
5-6 times/wk	2	1	1	3	4	0
≥1 time/d	4	2	6	5	6	2
Ultraprocessed foods, n						
Never	0	1	0	0	2	1
<1 time/wk	8	3	7	8	6	3
1-2 times/wk	6	12	7	6	7	11
4-6 times/wk	3	2	2	2	2	0
1 time/d	2	1	1	0	1	1
2 times/d	0	0	0	0	0	0
Fats and oils, n*^a^*						
1 time/wk	3	2	3	4	1	1
2-3 times/wk	5	5	5	6	7	3
4-6 times/wk	6	6	4	3	5	6
1-2 times/d	5	5	5	4	4	4
≥3 times/d	0	0	0	0	1	1

^*a*^n = 1 missing as participant in the standard care group did not respond to the question.

Dietary intake of energy, macronutrients, and several micronutrients was not different at baseline or end of study between the study groups (Table 4). At the end of the study, women in the intervention group had a lower intake of total sugar (64.1 ± 25.7 g/d vs 89.9 ± 29.8 g/d; *P* = .012) and a higher intake of linoleic acid (13.7 ± 5.2 g/d vs 9.1 ± 4.0 g/d; *P* = .023) compared to the respective intakes in the standard care group.

**Table 4.** Macronutrient intake at baseline and end of study in the intervention and standard care groups

	Baseline		End of study	
	Standard care	Intervention	Standard care	Intervention
Energy, kJ	8559 ± 2808	9155 ± 2440	8680 ± 1442	8623 ± 1093
Protein, g	105.8 ± 35.2	115.5 ± 38.2	103.8 ± 37.1	105.8 ± 21.8
Total fat, g	93.4 ± 43.2	97.5 ± 37.8	91.6 ± 23.4	100.2 ± 20.4
Saturated fat, g	34.5 ± 19.6	31.0 ± 14.7	35.9 ± 11.1	30.3 ± 7.0
PUFA, g	13.9 ± 7.6	16.2 ± 7.6	11.4 ± 5.2	16.3 ± 6.6
MUFA, g	36.0 ± 18.3	43.8 ± 20.0	37.1 ± 12.2	45.9 ± 13.5
Carbohydrates, g	184.7 ± 53.2	197.5 ± 71.8	193.2 ± 59.9	171.7 ± 30.1
Sugars, g	81.1 ± 31.9	66.7 ± 21.5	89.9 ± 29.8	**64.1 ± 25.7** *^b^*
Added sugars, g	23.0 ± 25.4	16.0 ± 16.4	30.4 ± 29.4	18.7 ± 20.2
Dietary fiber, g	29.4 ± 14.9	29.3 ± 11.5	28.4 ± 13.7	29.1 ± 10.1
% protein	20.7 ± 4.5	21.4 ± 4.7	20.4 ± 6.0	20.9 ± 3.6
% total fat	39.1 ± 8.3	38.9 ± 9.2	39.0 ± 8.6	42.8 ± 5.3
% saturated fat	14.1 ± 4.6	12.3 ± 3.9	15.4 ± 4.5	13.2 ± 3.3
% carbohydrate	36.4 ± 8.0	36.3 ± 11.0	36.6 ± 8.9	33.1 ± 5.1
Linoleic, g	12.3 ± 7.0	13.6 ± 6.5	9.1 ± 4.0	**13.7 ± 5.2** *^b^*
ALA, g	1.9 ± 1.5	1.8 ± 0.8	1.2 ± 0.7	1.6 ± 0.8
Very-long-chain ω-3 fatty acids, mg*^a^*	151.9 (99.6, 380.2)	136.7 (50.0, 280.0)	91.0 (55.3, 222.1)	136.4 (64.3, 1102.3)
EPA, mg*^a^*	44.5 (18.3, 93.8)	25.0 (4.9, 82.0)	19.5 (9.5, 66.6)	20.9 (5.9, 416.8)
DPA, mg*^a^*	68.3 (38.3, 91.1)	33.1 (18.8, 103.6)	32.9 (24.6, 135.0)	60.2 (31.1, 162.8)
DHA, mg*^a^*	62.4 (12.6, 217.6)	49.2 (13.7, 131.2)	23.8 (6.8, 64.1)	38.3 (29.0, 522.6)

Data presented as mean ± SD.

Abbreviations: ALA, α-linolenic acid; DHA, docosahexaenoic acid; DPA, docosapentaenoic acid; EPA, eicosapentaenoic acid; MUFA, monounsaturated fatty acids; PUFA, polyunsaturated fatty acids.

^*a*^Presented as median (interquartile range).

^*b*^ *P* less than .05 vs standard care.

#### Plasma biomarker concentrations

At the end of the study, no between-group differences were observed for triglycerides (mean [95% CI] in intervention, 2.84 [2.22-3.46] mmol/L vs standard care, 3.40 [2.78-4.02] mmol/L) or glucose (intervention, 5.62 [5.11-6.13] mmol/L vs standard care: 5.76 [5.25-6.26] mmol/L) as shown in Table 5. There was also no between-group difference in the change over time for triglycerides (mean difference [95% CI] in intervention, +0.16 [−0.29 to 0.60] mmol/L vs standard care, +0.51 [0.03-0.99] mmol/L) or glucose (−0.11 [−0.61 to 0.38] mmol/L and +0.19 [−0.33 to 0.70] mmol/L).

**Table 5.** Nonfasting biomarker concentrations at baseline and end of study

Biomarkers	Intervention (n = 15)	Standard care (n = 15)
Triglycerides, mmol/L		
Baseline	2.66 (2.23-3.09)	2.93 (2.20-3.67)
End of study	2.84 (2.22-3.46)	3.40 (2.78-4.02)
Glucose, mmol/L		
Baseline	5.53 (4.88-6.19)	5.78 (5.03-6.53)
End of study	5.62 (5.11-6.13)	5.76 (5.25-6.26)

Data presented as mean (95% CI). Intervention group vs standard care group, all *P* greater than .05.

#### Birth outcomes

Maternal and infant characteristics are reported in Table 6. Mean (95% CI) gestational weeks at delivery was 38.6 (37.8-39.3) and 38.9 (38.5-39.2) weeks for the intervention and standard care groups, respectively (*P* = .469). One woman in the intervention group delivered a baby preterm (<37 weeks’ gestation) due to complications from preeclampsia. No infants were born preterm in the standard care group. The number of women who delivered with natural vaginal birth was similar across groups. Compared to the standard care group, twice the number of women in the intervention group delivered via cesarean delivery, and half were delivered via assisted vaginal birth. Birth weight was significantly higher in the standard care group than in the intervention group (mean difference [95% CI]: 479.5 [110.7-848.3] g; *P* = .013). In the standard care group, 3 infants were born with macrosomia (>4000 g) and 2 were considered large for gestational age. There was no macrosomia or large for gestational age cases identified in the intervention group. The median 5-minute APGAR score was 9 both for the intervention and standard care groups.

**Table 6.** Maternal and neonatal outcomes in the intervention and standard care groups

Maternal	Intervention (n = 18)	Standard care (n = 16)
Gestational age at delivery, wk	38.6 (37.8-39.3)	38.9 (38.5-39.2)
Delivery mode, n (%)		
Vaginal birth	12 (66.7%)	13 (81.3%)
Cesarean	6 (33.3%)	3 (18.8%)
**Neonatal**	**Intervention (n = 17)**	**Standard care (n = 16)**
Birth weight, g	3040.6 (2763.9-3274.9)*^a^*	3520.1 (3303.4-3759.3)

Data presented as mean (95% CI).

^*a*^ *P* less than .05 vs standard care.

## Discussion

This is the first study investigating the feasibility of a personalized dietary intervention that targets triglycerides, rather than glucose, for the dietary management of GDM. There was a modest recruitment rate and a high retention rate. Compared with the standard care group, women in the intervention group showed improved self-reported dietary behaviors and had a lower mean birth weight, but no difference in plasma triglycerides.

The dietary strategy for the intervention group was based on our theoretical dietary modeling study aimed at reducing triglycerides (26). The optimal strategy included reducing 50% of ultraprocessed food intake and increasing a combination of fruits, vegetables, nuts, and fish high in long-chain ω-3 fatty acids, which was the focus for the study intervention group. We found a third of women in the intervention group decreased their intake of ultraprocessed foods. Although this was only a modest proportion of women, favorable effects on reducing intake of ultraprocessed foods have been observed for overall diet quality (36) and nutritional biomarkers (37). In Australia (36, 38) and internationally (39-41), consumption of ultraprocessed foods is high, and is higher than the recommendations for limiting discretionary choices (42). Although guidance on how to limit these foods was discussed with women in the intervention group, they may have prioritized other food group changes. Moreover, some women reported increasing their intake of ultraprocessed foods. While we did not question women about their responses, this finding might be due to prior, limited knowledge of types of ultraprocessed foods, leading women to potentially underreport at baseline or to be more accurate at the end of the study.

Many women in the intervention group increased their intake of nuts, by about half a handful per day, compared to the standard care group. Nuts are known for their cardioprotective effect, and an additional one serving per day has been shown to reduce the risk for chronic conditions such as coronary heart disease and cardiovascular diseases (43). Along with protein and fiber, nuts are high in linoleic acid, which we too found to be higher in the intervention group. Linoleic acid might contribute to the protective effect of nuts by reducing dyslipidemia, mainly through reducing cholesterol and increasing high-density lipoprotein cholesterol (44). Risk for these adverse cardiometabolic and cardiovascular health outcomes are further increased in women who have GDM (2, 45, 46), highlighting the potential effect that increasing intake of nuts has for these women. Although there were limited changes in fruit intake, most women were already consuming adequate intakes at baseline. Thus, the personalized dietary advice was focused more on maintaining their intake. We did not observe expected changes in vegetables or a change in saturated to unsaturated fats. Thus, a short-term, low-intensity dietary intervention might be insufficient to change all dietary behaviors at once, and more intensive dietary strategies may be required in future studies.

Notwithstanding the positive dietary changes achieved in the intervention group, the effect on triglycerides was negligible. There is no consensus as to the typical increase in triglycerides over the course of pregnancy, with only a few studies having been undertaken to elucidate this (17). Interestingly, the intervention group did demonstrate a smaller, albeit, nonsignificant, increase in triglycerides compared with the standard care group (0.16 mmol/L [6.8%] vs 0.51 mmol/L [16.7%], a change of 0.35 mmol/L [31 mg/dL]). The effect of this difference in pregnancy remains unclear, and we cannot be certain that the lower birth weight in the intervention group was a result from this smaller, attenuated rise in triglycerides. Other factors such as maternal BMI may also influence fetal growth (47). Although we did not conduct statistical testing of baseline characteristics following CONSORT recommendations for randomized trials (48), maternal BMI may have differed between groups by chance due to the small sample size. It is possible that this may have contributed to the higher birth weight observed in the standard care group. Key maternal characteristics such as prepregnancy BMI should be considered in future adequately powered trials to ensure balanced groups and to estimate the intervention effect more precisely on outcomes such as birth weight.

Overall, our results provide evidence that women in the intervention group adopted some important changes to their diet and were satisfied with and adhered to the dietary strategy aimed at attenuating the rise in triglycerides. The dietary outcomes provide a level of confidence that the null results for a change in triglycerides are not due to poor intervention delivery but rather due to other factors, for example, dietary change of this magnitude lacking any effect on plasma biomarkers, or perhaps a lack of statistical power. Future, powered RCTs are warranted to examine how dietary strategies, specifically aimed at attenuating triglycerides, can reduce fetal overgrowth in women with GDM.

### Strengths and Limitations

A key strength of this feasibility study was the randomized controlled design, which directly compared an active intervention group with a standard care group, minimizing selection bias. Recruitment targets were met, and the study had a higher retention rate. The dietary intervention was delivered through personalized dietary advice, following clear protocols, which following future larger trials could be readily translatable to clinical practice. The low-intensity dietary intervention embedded within routine antenatal care, and the telehealth dietary sessions, may also have encouraged participant adherence and retention in this trial.

Limitations include that assessment of maternal and neonatal outcomes are exploratory, with limited power to draw conclusion on the treatment effect. This trial was not blinded due to the nature of dietary interventions. Although unblinded studies may introduce “expectation bias” to the standard care group, the personalized nature of this dietary intervention will have minimized some of this risk. Researcher bias was mitigated by objective outcome assessments and a standardized data collection protocol. We did not measure changes in medication or insulin use in this feasibility trial, which is a limitation toward understanding the effectiveness of the diet in GDM management (16). Dietary recalls are subject to several types of bias (49) and the use of a one-off 24-hour recall at each study time point may not reflect a typical diet, especially when certain food groups, such as fish, were advised to be consumed several times a week rather than daily. Nevertheless, a single 24-hour recall was chosen to encourage participant recruitment and retention as other data collection methods can be burdensome and may discourage clinical trial participation (50).

### Conclusion

With a modest recruitment rate and high retention rate, this novel, personalized dietary intervention targeting triglycerides is feasible and acceptable for women diagnosed with GDM. The positive improvements observed in maternal nutritional intake and desirable birth weight warrant further investigation in a large, definitive, RCT.

## Data Availability

Data are available on request from the authors.

## Clinical Trial Information

Clinical trial registration number ACTRN12623000199617p (registered February 24, 2023).

## Abbreviations

BMI: body mass index
GDM: gestational diabetes mellitus
IQR: interquartile range
RCT: randomized controlled trial
RR: relative risk
